# Heart Failure Beyond the Diagnosis: A Narrative Review of Patients’ Perspectives on Daily Life and Challenges

**DOI:** 10.3390/jcm13237278

**Published:** 2024-11-29

**Authors:** Michelle Shigi Yang, Mohamed Bilal Abdallah, Zubair Bashir, Wissam Khalife

**Affiliations:** 1 Department of Cardiology, University of Texas Medical Branch, Galveston, TX 77555, USA; 2 Department of Health and Human Performance, University of Houston, Houston, TX 77004, USA

**Keywords:** heart failure, young adults, guideline-directed medical therapy, exercise

## Abstract

Heart failure (HF) is a complex syndrome that significantly affects patients’ physical, psychological, and socioeconomic well-being. Despite advances in guideline-directed medical therapy (GDMT), such as ACE inhibitors, beta-blockers, and SGLT2 inhibitors, HF continues to have a high global burden, with over 64 million affected worldwide and a five-year mortality rate of 50%. HF disrupts various life aspects, especially for younger patients (aged 35–55), who often face career interruptions due to severe symptoms like fatigue and frequent hospitalizations. These patients may struggle to maintain employment, resulting in financial instability compounded by high healthcare costs. Moreover, reduced exercise capacity and sexual dysfunction negatively impact patients’ quality of life. The psychological toll of HF is profound, with many patients experiencing depression, anxiety, and stress. However, a positive mindset has been shown to improve survival rates, underscoring the need for holistic management approaches. Interventions like cognitive behavioral therapy (CBT) and remote monitoring technologies such as CardioMEMS offer promising avenues to improve quality of life and reduce hospitalizations. This review highlights the importance of a multidisciplinary, patient-centered approach to HF management. Tailoring care to align with individual goals, integrating psychosocial support, and enhancing patient education are vital in addressing both the clinical and personal challenges of HF. By adopting a comprehensive approach, healthcare providers can significantly improve long-term outcomes and quality of life for HF patients.

## 1. Introduction

Heart failure (HF) is a complex medical syndrome characterized by compromised tissue perfusion due to the inability of the heart to generate adequate cardiac output (CO) and/or congestion due to abnormalities in the myocardial relaxation. It is caused by different etiologies that include ischemic heart disease, metabolic heart disease, infiltrative heart disease, structural heart disease, and heart disease caused by infections and drugs. It usually manifests with myriad symptoms, which commonly include dyspnea at rest or exertion, orthopnea, paroxysmal nocturnal dyspnea, weight gain, cough, wheezing, lower extremity edema, ascites, generalized fatigue, and weakness. In the United States, it is responsible for one-third of all the deaths attributed to cardiovascular disease and also has a high 5-year mortality rate of 50% [1].

The global prevalence of heart failure (HF) is estimated to be 1–2% in the adult population, affecting over 64 million individuals worldwide [2]. In the U.S., the prevalence stands at 2.3%, while in Europe, it is 1.7%. Incidence rates vary, with 1 to 9 cases per 100 person-years reported in the U.S. and 3.2 cases per 100 person-years in Europe [3]. Notably, the average age at HF diagnosis is steadily decreasing, and the incidence among adults under 50 years is rising, with this group now accounting for 10% of the overall HF population [4]. A Swedish study examining national hospital discharge and death registries between 1987 and 2006 demonstrated an increase in the incidence rate of HF by 50% and 43% in the age groups 18–34 and 35–44, respectively [5].

Furthermore, the Get With The Guidelines-Heart Failure (GWTG-HF) registry reported the 5-year mortality rate of patients hospitalized for HF between 2005 and 2009 to be 75% [3], and the age-adjusted mortality rate among the young adults increased by 5% each year between 2019 and 2022 [6]. However, the mortality rates between HFrEF and HFpEF of those hospitalized for HF between 2005 and 2009 were roughly equal at 75.2% and 75.7%, respectively [3]. While the exact cause of this rising trend among young adults remains uncertain, it is likely influenced by the global increase in heart disease risk factors, such as hypertension, hyperlipidemia, obesity, metabolic syndrome, diabetes, arrhythmias like atrial fibrillation, and the rising prevalence of substance abuse [7].

**Figure 1:**
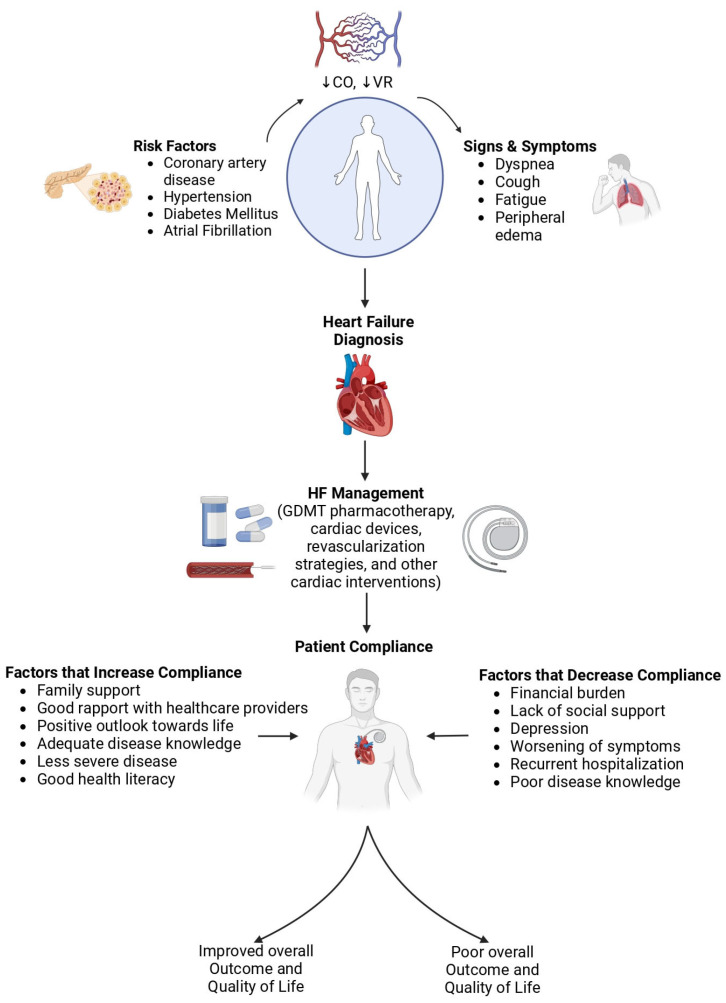
Central illustration. Comprehensive overview of heart failure diagnosis, management strategies, and key predictors of patient adherence to treatment plans. The figure was created with https://www.biorender.com/.

Beyond medical challenges, young adults with HF face profound impacts on their social lives, mental health, and future planning. Physical limitations interfere with careers, education, and social activities, often leading to significant emotional distress [8,9]. Many young adults experience anxiety and depression, worsened by the uncertainty of managing a progressive illness at a young age. Additionally, the financial burden of ongoing treatments, medications, and hospitalizations can be particularly difficult [10,11], especially for young adults as they often lack stable financial resources or comprehensive health insurance [6] (Figure 1).

Together, these challenges present a significant knowledge gap on the broader impact of HF on the lives of young adult patients who may face unique challenges. This review aims to address this gap by exploring the social, economic, and psychological effects of HF on the experiences of younger adults.

## 2. Classifications and Etiologies of Heart Failure

Many frameworks for HF classification exist. The New York Heart Association (NYHA) functional class classifies HF based on symptom severity and its impact on daily activities, dividing it into four classes. Class I has no limitation of ordinary physical activity, Class II has slight limitation of ordinary physical activity, Class III shows marked limitation in performing less than ordinary physical activity, and Class IV is when symptoms occur at rest or with minimal physical activity.

The American College of Cardiology Foundation/American Heart Association (ACCF/AHA) classifies HF into four stages based on the presence of signs and symptoms of HF and myocardial structural changes. Stage A refers to patients who are at high risk for HF but do not have underlying structural heart disease or symptoms of HF. These patients often have multiple risk factors, such as diabetes and hypertension, that predispose them to developing heart failure. Stage B includes patients with structural heart disease without signs or symptoms of HF, who are known as pre heart failure; Stage C consists of patients with structural heart disease and prior or current symptoms of HF; and Stage D is refractory HF requiring advanced HF therapy [12].

HF is also classified into four major phenotypic groups based on the left ventricular ejection fraction (LVEF) [12]. These include heart failure with reduced EF (HFrEF), in which the LVEF is ≤40%, heart failure with mildly reduced ejection fraction (HFmrEF), which has an LVEF between 41 and 49%, and heart failure with preserved ejection fraction (HFpEF), in which the LVEF is ≥50%. In addition, a group that shows an improvement in their ejection fraction from ≤40% to >40% and an absolute increase of ≥10% is identified as heart failure with improved ejection fraction (HFimpEF) (Table 1). HF can also be classified by etiology, with these underlying causes common across the various HF subtypes and classification systems mentioned above. The most common etiologies are ischemic (caused by critical stenosis of coronary artery) and non-ischemic cardiomyopathies (NICMs) (no significant coronary artery disease to explain the signs and symptoms of HF and the structural changes in the myocardium). NICMs include valvular, hypertensive, infiltrative, genetic, congenital, metabolic, and idiopathic (unknown etiology) causes [13].

## 3. The Patient’s Perspective

### 3.1. Economic Burden

The economic burden of HF on global healthcare systems is substantial and is expected to rise due to the increasing prevalence of the disease. A comprehensive study estimated that the worldwide economic costs of HF in 2012 totaled approximately USD 108 billion [10]. Of this, direct costs constituted about 60% (USD 65 billion), while indirect costs accounted for the remaining 40% (USD 43 billion) [10]. A systematic review analyzing 16 international studies reported an average lifetime healthcare cost for HF per person to be USD 126.819 between 2004 and 2016 [14]. The cost of HF management is further increased by the presence of comorbidities, such as presence of type 2 diabetes mellitus, which showed an average increase in HF management expenditure per annum from USD 22,230 to USD 32,676 in 2010 [15].

The analysis of insurance payment trends for HF patients from 2006 to 2021 reveals variations across age groups and insurance types. Commercial insurance costs rose significantly from 2006 to 2017, suggesting that younger adults, often covered by employer-sponsored plans, faced increasing financial burdens. Costs then leveled off slightly from 2017 to 2021. Managed Medicaid saw a modest decline in costs, reflecting cost-management efforts, while self-insured plans had a gradual increase, indicating rising financial pressures on employers and employees [16].

Medicare risk payments steadily increased, likely due to higher utilization rates, whereas Medicare cost plans showed significant fluctuations, including a sharp decline from 2015 to 2018. This can be attributed to the implementation of the Medicare Access and CHIP Reauthorization Act (MACRA) in 2015, which may have benefited Medicare patients disproportionately at the expense of younger, non-Medicare patients [16,17].

Younger heart failure patients, particularly those under 50, face growing out-of-pocket expenses, emphasizing the need for insurance reforms and targeted support programs.

A bimodal pattern in average medical costs emerged, with declining trends in those over 65 years, most notably in individuals over 85 (AAPC −3.66%, 95% CI: −5.43% to −1.99%). Conversely, younger adults under 50 years experienced the steepest rise in costs, with an AAPC of 7.35% (95% CI: 5.07% to 9.61%), highlighting an increasing financial strain on younger HF patients, which is in line with the above-mentioned insurance expenditure [16].

### 3.2. Financial Burden on the Individuals

A recent analysis of Medicare drug plans [17] revealed that the average 30-day out-of-pocket cost for the recommended four different drug classes of goal-directed medical therapy (GDMT) ranged from USD 84 to USD 100, with a median of USD 94. This was primarily driven by the costs of angiotensin receptor neprilysin inhibitors (ARNis) and sodium-glucose cotransporter-2 inhibitors (SGLT2is). The four pillars of guideline-directed medical therapy (GDMT) have demonstrated mortality and morbidity benefits in patients with HFrEF and carry a Class 1A recommendation [12]. However, there is evidence of benefit with aldosterone antagonists and SGLT-2 inhibitors in HFpEF, which hold Class 2B and 2A recommendations, respectively [12]. Nonetheless, according to a nationally representative cross-sectional study using the Medical Expenditures Panel Survey (MEPS), one in three adults with HF in the US reported subjective financial hardship from medical bills [18]. Moreover, 20% of patients reported problems with paying medical bills, while 13% expressed an inability to pay their bills at all. This is more alarming for people under the age of 65, as 26% reported issues with paying medical bills and 24% expressed an inability to pay medical bills at all [19]. Additionally, a prior study involving HF patients revealed that 40% of patients with an annual income greater than USD 100,000 would be unlikely to take medication costing USD100 per month due to the rising out-of-pocket costs across all the income strata [19].

AHA/ACC/HFSA Guidelines recommend initiation of GDMT and up-titrating the medications to maximum tolerated dosages in HFrEF patients [12]. However, the initiation of different classes of medications may be limited by affordability issues, especially among the young adults who may have fewer comorbidities than their older counterparts. When comparing the CHECK-HF and CHAMP-HF registries, which represent the Dutch and USA HF patient populations, respectively, a higher percentage of the Dutch cohort were on optimal GDMT compared to the USA cohort, despite having an older population with worse kidney function [20,21]. Specifically, a higher percentage of Dutch HF patients were on loop diuretics (81% vs. <30%), RAAS inhibitors (84% vs. 73%), beta-blockers (86% vs. 67%), and MRAs (56% vs. 33.4%) [20,21]. Additionally, fewer than 30% of patients in the US cohort were on target doses of RAAS inhibitors and beta-blockers [21]. It is speculated that the difference in healthcare systems may be influencing the disparities in the use of GDMT between the two countries. The Netherlands government mandates healthcare insurance for all citizens and partially pays for the healthcare [22]. However, in the USA, approximately 7.7% of the population is uninsured in 2024, which may limit the initiation and maintenance of many of the GDMT medications [23]. The ASIAN-HF global survey found differences in adherence to GDMT based on geographical region, highlighting possible differences in insurance and government policies, provider education, culture, and the population attitude towards medical care [24]. The ASIAN-HF study included 46 centers from 11 countries in Asia, with mean age of 61.6 ± 13.3 years and 27% women representation. It reported marked variations among countries in the use of GDMT, with Indonesia having the lowest usage (61.7%) of β-blockers but the highest usage of ACEIs/ARBs (85.8%), whereas Japan had highest usage of β-blockers (89.5%) but moderate use of ACEIs/ARBs (78.4%). Moreover, Indonesia and the Philippines had the highest mortality rates at 21.4% and 14.3%, respectively, despite having the youngest populations. In contract, Japan, with a relatively elderly population, had the lowest 1-year mortality rate at 4.4% [24]. This shows that HF is adversely affecting the young population and there are factors beyond the prescription of medications that influence the HF outcomes in young adults.

Moreover, there is a significant financial burden associated with the management of HF for individual patients and their families. The total annual cost spent by individuals is estimated to range from USD 868 for South Koreans to USD 25,532 for Germans. The major chunk of this cost is due to HF hospitalizations followed by medication expenses, which may also limit the initiation and maintenance of GDMT among HF patients [14].

### 3.3. Barriers to Adherence and Implementation of Heart Failure Management Strategies

Various factors are reported to limit the initiation and maintenance of GDMT. Factors such as local hospital protocols, clinicians’ comfort in initiating these therapies, and differences in the care settings contribute to the disparities in the use of these medications [25]. Moreover, it is noted that the sickest and the frailest patients are often on less intensive GDMT treatment, most likely due to the physician’s concern for drug-related adverse events [26]. While advanced HF patients are less likely to tolerate GDMT due to hypotension, low cardiac output, and severe kidney dysfunction, the proper implementation of GDMT is associated with better prognosis [27].

A significant difference was found in the prescription of GDMT between family medicine/internal medicine and cardiology practices. BB prescription was found to be significantly lower among internal medicine and family medicine physicians as compared to cardiologists (42.3% vs. 70.5%) [26]. In addition, a secondary analysis of the GUIDE-IT trial revealed that only 15.5% of patients were on the optimal guideline-directed therapy at 6 months [28].

Social determinants of health, which may encompass patient access to transportation and also the inability to take time off due to the nature of work, may contribute to no shows at appointments requiring frequent blood work to monitor the disease status and the side effects of the medications.

Moreover, perceived social support (PSS) and medical adherence are independent predictors of event-free survival rates in HF patients [29]. A study reported an inverse relationship between perceived social support and both readmission rates and cardiovascular mortality. Patients with greater perceived social support had a significantly lower risk of all-cause admission (OR = 0.966; 95% CI = 0.944–0.988; *p* = 0.003) and cardiac readmission (OR = 0.970; 95% CI = 0.949–0.992; *p* = 0.008) [30]. Supporting this finding, another study showed that heart failure patients who are single and lack social support have twice the risk of hospital readmission or death (aHR 2.1, 95% CI 1.3–3.3) after adjusting for demographics and clinical profiles [31].

A qualitative study using semi-structured interviews revealed that patients viewed adherence as a spectrum, which include adapting recommendations to their individual circumstances, and considered hospital readmission a rational choice rather than a negative outcome [32], which potentially increases the readmission rates.

**Figure 2:**
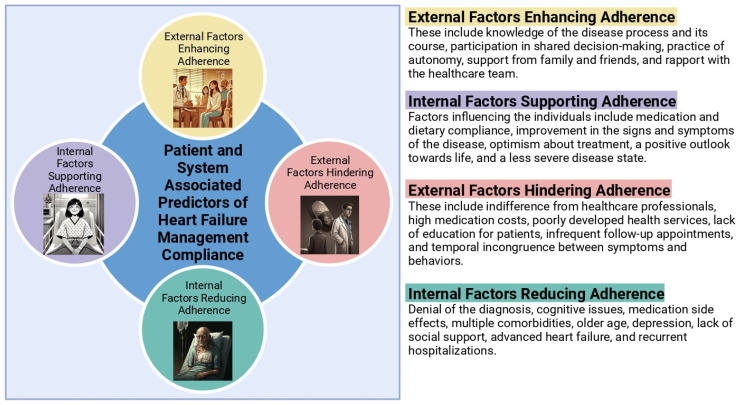
Patient- and system-associated predictors of compliance with heart failure management. The figure was created with https://www.biorender.com/.

Several factors contributed to patient non-adherence, including misalignment between behaviors and symptoms, uncertainty about recommendations, socioeconomic status, comorbidities, and feelings of hopelessness. Additionally, lack of capacity to provide adequate patient education and an appropriate follow-up visit schedule, in addition to poorly developed health services, may also contribute to decreased patient adherence [8]. Furthermore, Sevilla-Cazes et al. suggested that HF patients should be managed holistically, with recommendations extending to include pain management, psychological, spiritual, social support, and complex care coordination [32]. Positive predictors of compliance included a higher EF, while negative predictors included age over 65, lack of family support, NYHA class of III or IV, number of hospitalizations, and side effects of medications [33]. Factors that improved compliance included health improvement, adaptation to HF, optimism regarding treatment strategies, and a positive outlook towards life, as well as access to information, a sense of autonomy with respect to management, a good rapport with the physician, and social support. Conversely, denial, forgetfulness, high cost of treatment, side effects of medications, and lack of healthcare support decreased compliance [34] (Figure 2).

Despite the strong recommendations for GDMT in HFrEF, large registries have reported poor utilization, even before the introduction of SGLT-2 inhibitors as standard GDMT [24,35]. For example, only 1.1% of patients in the CHAMP-HF registry received triple therapy at target doses for renin–angiotensin–aldosterone system (RAAS) inhibitors, beta-blockers, and mineralocorticoid receptor antagonists [21]. The 2018 ASIAN-HF registry consisting of 5000 patients with HFrEF and stage C HF showed a low prevalence of GDMT, with only 17% of patients on ACEis, 13% on BBs, and 29% on MRAs [24]. Moreover, the QUALIFY international survey collected data from 547 centers across 36 countries in Africa, Asia, Australia, Europe, the Middle East, and the Americas, with a mean patient age of 63.1 ± 12.5 years and 26% female representation. It found that the dosing of recommended medications was often suboptimal: only 27.9% of patients on ACE inhibitors and 14.8% on beta-blockers received target doses as per international guidelines. Cardiac resynchronization therapy (CRT) was used in just 9% of patients, with significant regional variation (Western Europe 19.6%, Central/Eastern Europe 4.7%, Asia 7.9%, and other regions 8.9%; *p* < 0.001). Similarly, implantable cardiac defibrillator (ICD) use was low overall at 9.7%, with rates differing by region (Western Europe 26.4%, Central/Eastern Europe 5.4%, Asia 3.4%, and other regions 7.8%; *p* < 0.001) [36].

Analyses of data from several HF registries have revealed suboptimal implementation of guideline-directed medical therapy for heart failure. While prescription rates for RAAS inhibitors, beta-blockers, and mineralocorticoid receptor antagonists were relatively high, ranging from 73.4% to 92.6%, 67.0% to 93.3%, and 33.4% to 74.5%, respectively, the rates of achieving target doses were much lower. For RAAS inhibitors, the target dose achievement rates were 16.3% to 43.6%, with the specific breakdown rates for ACE inhibitors, ARBs, and ARNIs of 15% to 27.9%, 6.9% to 23.3%, and 30%, respectively. For BBs and MRAs, the target dose achievement rates ranged from 12% to 27.5% and 23.5% to 76.6%, respectively [20,25,37,38].

These registries suggest that older age and frailty are the primary barriers to GDMT initiation and up-titration, as there is poorer adherence in elderly patients with hypotension, impaired renal function, and comorbidities [20,21,38]. Additionally, patient adherence to prescribed GDMT therapies is often suboptimal, which may be attributed to a perceived lack of medication efficacy, mental health conditions, and inadequate health literacy [32].

### 3.4. Impact of Heart Failure on Career, Education, and Financial Stability

HF can negatively impact patients’ daily lives and occupational careers due to symptom burden and frequent hospitalizations. A study involving semi-structured interviews with young HF patients aged 35 to 55 found that they experienced career interruptions, ranging from continued work with a high symptom burden to disability [9]. Some participants also reported difficulty continuing their educational goals due to illness severity [9]. Many patients cited professional success as a source of pride; however, career disruptions due to symptom burden and the associated psychological and social effects resulted in a loss of this pride and problems with self-identity [9].

Furthermore, younger HF patients often struggle to maintain a career or achieve financial security, owing to the high costs of managing numerous comorbidities associated with HF [9]. A cross-sectional study involving participants with a mean age of 56 years revealed that 42% of HF patients were receiving disability compensation, and 63% mentioned that fatigue interfered with their normal work [39].

### 3.5. Impact on Psychological Well-Being

HF diagnosis can have a detrimental impact on psychological health, leading to depression, stress, and anxiety, which can further worsen compliance and influence patient outcomes. A nationwide study of US veteran men found that patients with HFrEF and a history of post-traumatic stress disorder had a higher rate of coronary artery disease and had higher all-cause mortality compared to those without PTSD (73% vs. 64%) [40]. Additional stressors include frequent clinic visits, hospitalizations, testing, and polypharmacy, which also contribute to the economic burden on HF patients. This financial strain can exacerbate stress and have adverse effects [32]. A study with 149 HF patients found that the average cost of healthcare was between USD 3913 and USD 5829, and 54% of the patients were unable to pay for their monthly expenses [41]. Hence, in addition to the financial hardships affecting HF patients, they also face difficulties in routine activities like work, sports, and family time [8]. These factors may lead to depression, medication non-compliance, and worsening of the HF symptoms [8]. In contrast, a positive mindset has been associated with longevity and better health outcomes in HF patients [34]. A population-based observational study consisting of about 2 million people revealed that depression among HF patients is associated with an increase in HF progression by 18% in 7 years, after adjusting for cardiovascular risk factors [42]. In contrast, having a positive mindset can lead to longevity and better health [34]. Moreover, HF patients with a positive mindset revealed lower levels of pro-inflammatory cytokines and a lower mortality rate [43]. A review by Sin et al. showed the same result with a reduction of 35% in mortality risk over 5 years among HF patients with a positive mindset compared to those with a negative mindset [44].

### 3.6. Changes in Physical Activity Level

The physical and sexual activity of HF patients is limited due to a myriad of symptoms including generalized fatigue and weakness. In addition, these patients also spend more days in the hospital as compared to the general healthy population [41,42]. Greater severity of symptoms such as fatigue and weakness is accounted for when staging the level of disease in the NYHA classification system [13]. Patients with HF report having trouble climbing stairs, walking, and standing as they experience dizziness and fatigue [45]. About 60% of HF patients report having sexual problems [46]. These usually arise from low libido, fear of underperformance during sex, difficulties with orgasms, and erectile dysfunction in men. HF patients were reported to have higher rates of erectile dysfunction as compared to non-HF patients (37% vs. 17%) [47]. Additionally, younger patients and patients with a partner reported increased sexual dissatisfaction compared to their healthy counterparts and sexual dysfunction was more commonly seen in men and with a prescription of beta-blockers [47]. Physical activity patterns and intensity differ significantly between patients with HFrEF and those with HFpEF. A study comparing the level of activity reported that patients with HFrEF tend to be more physically active than those with HFpEF, showing notably higher daily step counts. Additionally, HFrEF patients were found to engage in more moderate-to-vigorous physical activity each day and have more frequent episodes of such activity than HFpEF patients [48]. This may suggest that HFrEF patients may have a higher baseline level of physical resilience or fewer limitations in daily activity compared to HFpEF patients.

However, exercise training can significantly improve exercise capacity in both HFrEF and HFpEF patients. It can also improve the LVEF and BNP/NTproBNP in HFrEF patients, whereas it can enhance diastolic function in HFpEF patients [49].

### 3.7. Changes in Sleep

Disturbance in sleep pattern has been reported among HF patients and also carries a significant morbidity and mortality burden [50]. Obstructive sleep apnea (OSA) is found in 20% to 60% of HF patients [51]. HFrEF are reported to have higher prevalence of sleep-disordered breathing (SDB) (47–81%) and OSA (12–53%) as compared to the general population [52]. In addition, the incidence of central sleep apnea (CSA) is reported to be 40% among HF patients [53]. CSA can activate the sympathetic nervous systems due to an imbalance in myocardial oxygen demand and supply leading to left ventricle overload and, hence, exacerbating HF. OSA also impacts quality of life with more frequent HF hospitalizations, and has also been shown to increase mortality [51]. Furthermore, sleep disturbances are associated with psychological disorders such as depression, worsening fatigue, increasing drowsiness, and poor concentration during day time. They also lead to arrhythmia and uncontrolled HTN; these are well-established risk factors for HF exacerbation [52,54].

### 3.8. Heart Failure Hospitalization Burden

HF is one of the common causes of hospitalization in the U.S, constituting over 1 million hospitalizations annually [55]. In 2018, 1,135,900 patients were hospitalized for HF, compared to 569,000 for COPD and bronchiectasis [56]. A 2019–2020 study of 263 HF patients (mean age: 51.1 ± 19.2 years) found an average hospital stay of 17.3 ± 7.3 days [57]. In contrast, the average length of stay for osteoarthritis was only 2 days [56], whereas the mean length of hospital stay for the general population was 5.5 days in 2018 [58]. In addition, a study with 146 HF patients reported 42.1% admissions due to non-adherence to pharmacotherapy (*p* = 0.02) [59]. This illustrates a significant burden of HF hospitalizations compared to other chronic illnesses, contributing to rising morbidity and mortality, especially among young adults.

### 3.9. Limitations

This review has several limitations. First, while we discuss the social, psychological, and financial impacts of HF on young adults, we provide only a broad overview rather than an in-depth analysis of each aspect. Each of these topics could be explored extensively in a dedicated review, yet our objective here was to offer a comprehensive overview of how HF affects young adults beyond its medical dimensions. Second, this is a narrative rather than a systematic review, and we did not restrict our sources by a specific time frame. This approach allowed for a broader perspective but may introduce selection bias, as we did not systematically limit or prioritize certain types of studies. Third, this review may not fully capture the diverse social and cultural factors that shape adherence, coping, and outcomes for HF patients. As a result, some findings may not fully apply to populations with different social supports, cultural perspectives on healthcare, or varying levels of health literacy.

## 4. Future Directions

To enhance health outcomes and quality of life (QoL) for heart failure (HF) patients, future efforts should focus on developing personalized care strategies. Tailoring treatment plans to align with each patient’s goals, values, and preferences will be essential for fostering adherence and engagement. Clinicians should prioritize understanding whether patients aim for longevity, symptom reduction, or independence, as this alignment can significantly impact treatment effectiveness [60]. Additionally, integrating psychosocial support programs, including stress management and cognitive behavioral therapy (CBT), is crucial for addressing the mental health challenges commonly faced by HF patients. Research indicates that such interventions can effectively reduce depression and anxiety, thereby improving overall QoL [61]. Empowering patients through informed decision-making and emphasizing patient education will further enhance self-care practices, motivation, and mental well-being [62].

Utilizing advanced remote monitoring technologies like CardioMEMS should be prioritized for effective HF management, especially among patients with recurrent admissions. These tools not only help in reducing readmission rates but also provide critical hemodynamic data for remote patient management [63]. Furthermore, the integration of palliative care alongside conventional HF treatment is recommended, as studies have demonstrated that a person-centered approach combining active management and supportive care can improve QoL for HF patients [64]. In summary, a comprehensive strategy that emphasizes personalized care, patient autonomy, educational initiatives, psychosocial support, and technological integration will be vital for improving the quality of life and overall well-being of patients living with HF in the future.

## 5. Conclusions

HF extends beyond its pathophysiological effects, profoundly impacting various aspects of a patient’s life. The psychological and financial burdens associated with HF cannot be overstated, and alleviating these burdens requires a collaborative effort. While patient adherence to medication is crucial, it is equally important for family members and healthcare providers to share the responsibility of managing the disease’s progression.

Effective management begins with the appropriate implementation of GDMT alongside thorough education for both patients and their families about supportive strategies. These measures can significantly enhance QoL and survival outcomes. Physicians play a pivotal role not only in administering GDMT but also in establishing a strong rapport with patients, particularly those managing chronic conditions. Educating patients about their disease and the consequences of non-adherence can lead to better compliance.

Additionally, engaging interdisciplinary teams is vital for comprehensive support. Physical therapists can assist in developing safe and effective exercise regimens, while social workers can help navigate financial and logistical barriers related to management strategies. Counseling services offer essential emotional support, normalizing the challenges of living with a chronic illness.

Finally, hospital administration can contribute by tracking high-risk patients and implementing strategies to reduce healthcare costs. Ultimately, the management of HF is a collective effort, necessitating a team approach where all facets of healthcare work in harmony to support patients on their journey to better health. This multidisciplinary collaboration is essential for enhancing patient outcomes and overall well-being.

## Figures and Tables

**Table 1:** Heart failure classification systems by ejection fraction, American Heart Association stages, and New York Heart Association functional class.

Criteria	Category	Definition
**Classification based on different Phenotypic presentations**		
**Ejection Fraction (EF)**	Heart Failure with Reduced EF (HFrEF)	LVEF ≤ 40% (Reduced pumping function)
	Heart Failure with Mildly Reduced EF (HFmrEF)	LVEF 41–49% (Mildly reduced pumping function)
	Heart Failure with Preserved EF (HFpEF)	LVEF ≥ 50% (Normal pumping function)
	Heart Failure with Improved EF (HFimpEF)	LVEF improved from ≤40% to >40%, and absolute increase of ≥10%
**Classification based on Signs, Symptoms, and Myocardial Structural Changes**		
**AHA Stages**	Stage A	At risk for heart failure, no structural heart disease or symptoms
	Stage B	Pre heart failure. Structural heart disease, but no symptoms
	Stage C	Structural heart disease with prior or current symptoms
	Stage D	Refractory heart failure requiring advanced therapies
**Classification based on Functional Capacity**		
**NYHA Classification**	Class I	No limitation of physical activity; no symptoms with ordinary activity
	Class II	Slight limitation; comfortable at rest, symptoms with ordinary activity
	Class III	Marked limitation; comfortable at rest, symptoms with less than ordinary activity
	Class IV	Symptoms at rest or with minimal activity
